# Artificial Intelligence in Neurology: Assessing the Diagnostic and Therapeutic Reasoning of Large Language Models

**DOI:** 10.7759/cureus.98570

**Published:** 2025-12-06

**Authors:** Skylar Bentley, Rees P Ridout, Nadiya A Persaud, Kevin M Sherin

**Affiliations:** 1 Research, Orlando College of Osteopathic Medicine, Winter Garden, USA; 2 Neurology, University of Central Florida College of Medicine, Orlando, USA; 3 Primary Care, Orlando College of Osteopathic Medicine, Winter Garden, USA

**Keywords:** artificial intelligence, clinical decision-making, guillain-barré syndrome, large language models, neurology

## Abstract

Background

Artificial intelligence (AI) and large language models (LLMs) are increasingly integrated into medicine, yet their role in clinical decision-making remains underexplored. Neurology provides an important testing ground for these systems because neurological diagnoses often involve complex, overlapping syndromes, subtle clinical distinctions, and time-sensitive decision-making that can significantly affect patient outcomes.

Objective

This study evaluated the diagnostic and therapeutic reasoning of three LLMs: ChatGPT 4.0, Google Gemini, and Claude 3.5 Sonnet, using a complex neurological case of acute Guillain-Barré syndrome (GBS) in the setting of chronic spinal epidural lipomatosis (SEL).

Methods

Each model was prompted with an identical case and instructed to generate both a diagnostic assessment and a treatment plan. Four blinded, board-certified neurologists assessed outputs using a standardized rubric across four domains: diagnostic accuracy, differential diagnoses, intervention plan, and monitoring/follow-up.

Results

All three models correctly identified GBS and proposed guideline-consistent therapies (IVIG, plasmapheresis). Claude 3.5 Sonnet achieved the highest mean total score (18.5/20), followed by ChatGPT (17.5) and Google Gemini (17.25). Domain-level scoring demonstrated distinct performance patterns: Google Gemini scored highest in primary diagnosis, Claude 3.5 Sonnet achieved the only perfect mean score in differential diagnoses and led in intervention planning, while ChatGPT performed strongest in monitoring and follow-up recommendations. Although all models produced clinically appropriate assessments and plans, none matched the physician’s gold standard in contextualizing SEL-specific considerations or developing detailed, longitudinal management strategies. These findings reflect descriptive reviewer assessments only; no inferential statistical testing was performed due to the study’s single-case design.

Conclusions

These findings highlight both the promise and limitations of LLMs in supporting complex neurological decision-making. While capable of accurate diagnosis and evidence-based interventions, LLMs demonstrated gaps in personalization and long-term care planning, underscoring the need for cautious integration alongside physician expertise.

## Introduction

In the past decade, Artificial Intelligence (AI) has been transforming medicine by assisting with clinical workflows and providing decision support for complex medical cases. Since 2019, particularly in the United States and China, there has been a marked surge in AI-related publications, including in the field of neurology [1,2]. Several routine applications now involve utilizing AI to help read diagnostic imaging. These include CT and MRI for neurovascular and neuro-oncologic cases, electroencephalogram (EEG) recording analysis and monitoring for seizure detection, stereotaxic image guidance for surgical spatial positioning, and quantification of symptoms in diagnosis and monitoring of movement disorders [3]. Neurology, in particular, represents a meaningful testing ground for AI because many neurological disorders involve complex presentations, overlapping symptom profiles, and time-sensitive diagnostic and therapeutic decision-making.

Large language models (LLMs) are examples of AI tools being used in medicine for diagnostic and therapeutic guidance. LLMs are pretrained on vast text corpora and can generate human-like responses to free-text prompts, including medical queries, without task-specific fine-tuning [4]. Common LLMs include ChatGPT, Google Gemini, and Claude 3.5 Sonnet. They also hold potential in improving patient education by expanding access to medical information to help patients understand their health conditions, prognosis, and therapeutic options [5]. However, there is currently a large gap in knowledge regarding the use of LLMs in patient care, and the diagnostic potential of these systems requires further evaluation in multifaceted clinical presentations. Although LLMs have shown impressive performance on medical reasoning examinations, prior studies have largely focused on board-style questions or straightforward clinical scenarios. Their ability to manage complex cases involving overlapping pathologies and comorbidities remains largely unexplored, and it is still unclear if these tools quantitatively improve physician diagnostic reasoning [6]. Because diagnostic errors are a substantial source of patient harm, this potential for improvement warrants further analysis. 

Contextual understanding is an essential component of the diagnostic process. LLMs have consistently displayed the ability to strictly adhere to preset diagnostic and treatment guidelines, but it remains unclear whether they possess the ability to go beyond applying guidelines and incorporate specific nuances, patient variables, and real-world complexity that can complicate a patient case [6]. An example of this contextual capacity involves the ability to integrate overlapping pathology (i.e., recognize how one condition can alter or obscure the standard presentation of another condition, making diagnosis more difficult). Further, such scenarios involve potentially modifying the standard management for a condition to account for comorbidities. 

This study involves a neurological case in which a patient presents with both Guillain-Barré syndrome (GBS) and spinal epidural lipomatosis (SEL). GBS typically presents as rapidly progressive, symmetric, ascending weakness with areflexia, often days to weeks after a preceding viral or gastrointestinal illness. SEL presents with signs of spinal cord or nerve root compression, including chronic back pain, neurogenic claudication, or progressive lower extremity weakness. This dual pathology was intentionally selected because it challenges both diagnostic reasoning (distinguishing overlapping neurological presentations) and therapeutic planning (adjusting interventions in the presence of comorbid disease), making it a rigorous test of LLM clinical applicability. This study aimed to determine how accurately and comprehensively three large language models can generate diagnostic assessments and treatment plans for a complex neurological case involving overlapping pathologies.

## Materials and methods

Case prompt development

A standardized case presentation was structured using a real-world case report [7]. The case consisted of a challenging neurological case of a patient presenting with acute GBS in the setting of chronic SEL. Patient history was based on a standard GBS presentation: recent viral illness with rapid progression of ascending paralysis with areflexia and sensory deficits. Notably, the patient’s concurrent SEL could cause a similar presentation, particularly in morbidly obese patients, which created a contextual challenge. The case was written in a clear and concise manner to avoid ambiguity. Because this study relied on a single standardized case, it should be considered an exploratory pilot study rather than a generalizable performance evaluation. No identifiable patient information was used, and the case content was derived exclusively from a previously published report; therefore, no additional ethical approval was required. This prompt served as the standardized input for all AI models.

Only information directly relevant to distinguishing GBS from SEL was included; comorbidities such as hypertension, diabetes, or hypothyroidism were not incorporated, as the intent was to isolate the diagnostic and therapeutic impact of overlapping neurological pathology rather than multisystem disease. Similarly, disease stage and severity were described only to the extent reported in the source case, and no additional modifying factors were added. No additional inclusion or exclusion criteria were applied beyond selecting a published case that demonstrated overlapping symptomatology, and these choices are acknowledged as methodological constraints.

LLM plan assessment and plan generation

Three LLMs were selected for evaluation based on their accuracy in medical reasoning: OpenAI’s ChatGPT 4.0, Google Gemini, and Claude 3.5 Sonnet. Each LLM was given an identical prompt of the patient case described above and instructed to produce two outputs. First, an assessment that included a diagnostic conclusion with other differential diagnoses and supporting reasoning for each. Second, a treatment plan with strategies for intervention, monitoring, and follow-up recommendations. The entire output from each LLM was saved for later evaluation. 

For transparency and reproducibility, all models were accessed via their respective web interfaces using default settings (temperature and system parameters not user-modifiable). Access dates were documented (April 2025 for all models). No API calls, custom prompts, or fine-tuning were used.

Physician’s plan

The assessment and management plan by the treating physician in the real-world case report was retrieved. It served as the reference standard for comparison when evaluating LLM plans. The plan included the physician’s diagnostic conclusion and comprehensive management strategy for the patient. This terminology was chosen because expert management may vary among clinicians.

Evaluation rubric development

A standardized scoring rubric was created to help with quantitative, objective comparison of LLM assessments and plans. The rubric consisted of four categories. First, diagnostic accuracy and reasoning are used to evaluate the accuracy of the diagnosis and its associated clinical reasoning and justification. Second, differential diagnoses are used to evaluate the capacity of the system in weighing alternative possibilities. Third, an intervention/treatment plan, to evaluate the appropriateness and level of detail provided for the intervention. Fourth, monitoring/follow-up care to evaluate the appropriateness of the recommended monitoring and follow-up strategies. Each category was scored using a Likert scale from 1 to 5, with specific descriptions dictating the reasoning for scoring a given point value. In general, the following standards of scoring were used: 5 = Detail-rich, clinically sound approach aligning with expert standards; 3 = Basic assessment and plan with notable gaps or omissions; 1 = Inaccurate or inappropriate plan. The maximum total score achievable for an LLM’s given assessment and plan was 20 points. The rubric was developed specifically for this study and was not formally validated, which we acknowledge as a limitation; future work should include rubric validation and assessment of inter-rater reliability.

Scores of 2 and 4 represented intermediate performance between these anchors and were used when an evaluator determined that an LLM’s output partially met criteria for the next highest score but did not fully satisfy it. Although this allowed flexibility, the absence of explicit descriptors for these values may introduce variability in scoring, which we acknowledge as a limitation.

The four rubric domains were selected because they represent widely accepted components of clinical reasoning in neurology: accurate diagnosis, thoughtful differential generation, evidence-based intervention planning, and safe monitoring/follow-up. These domains were intended to capture the core elements most relevant to evaluating LLM clinical decision-making; however, they may not encompass all nuances of real-world practice, such as prognostication, interdisciplinary coordination, or shared decision-making.

The rubric was developed specifically for this study and was not formally validated. Future work should include rubric validation and assessment of inter-rater reliability to strengthen content validity and consistency.

Reviewer assessment

Four board-certified neurologists, each with experience treating GBS patients, were recruited to evaluate LLM assessments and management plans. The physicians were blinded to which specific LLM generated each assessment and plan to reduce bias. They were provided the standardized scoring rubric described above, and they used it to quantitatively evaluate each LLM’s output. Their scoring focused on several aspects: the accuracy of the LLM’s final diagnosis (GBS) and differential diagnoses, ability to generate relevant differential diagnoses, appropriateness of the treatment plan and interventions (e.g., IVIG, plasmapharesis, respiratory support), as well as follow-up and monitoring strategies. A total score was generated for each LLM output by summing each of the four categories. All reviewer scores were completed independently; no consensus scoring process was used. Although reviewer scoring was independent and blinded, inter-rater agreement metrics (e.g., kappa, ICC) were not computed due to the limited number of reviewers and are identified as important future methodological additions.

Statistical analysis

Mean total scores (sum of rubric domains) and mean domain-specific scores (diagnostic accuracy, differential diagnoses, intervention plan, and monitoring/follow-up) were calculated for each LLM. Mean scores were calculated by averaging the four independent reviewers’ numeric ratings for each rubric domain, and total scores represent the sum of these domain-level means. Analyses were limited to descriptive statistics; no formal hypothesis testing was performed, and no p-values are reported. Analyses were limited to descriptive summaries only; no inferential statistics were performed. A formal measure of inter-rater reliability (e.g., κ statistic or ICC) was not calculated due to the small number of raters and the limited number of ordinal scoring categories. Future studies with larger reviewer cohorts should incorporate inter-rater reliability testing to strengthen reproducibility, as well as measures of variance. All analyses were conducted in R (R Foundation for Statistical Computing, Vienna, Austria; version 4.4.3).

## Results

**Figure 1:**
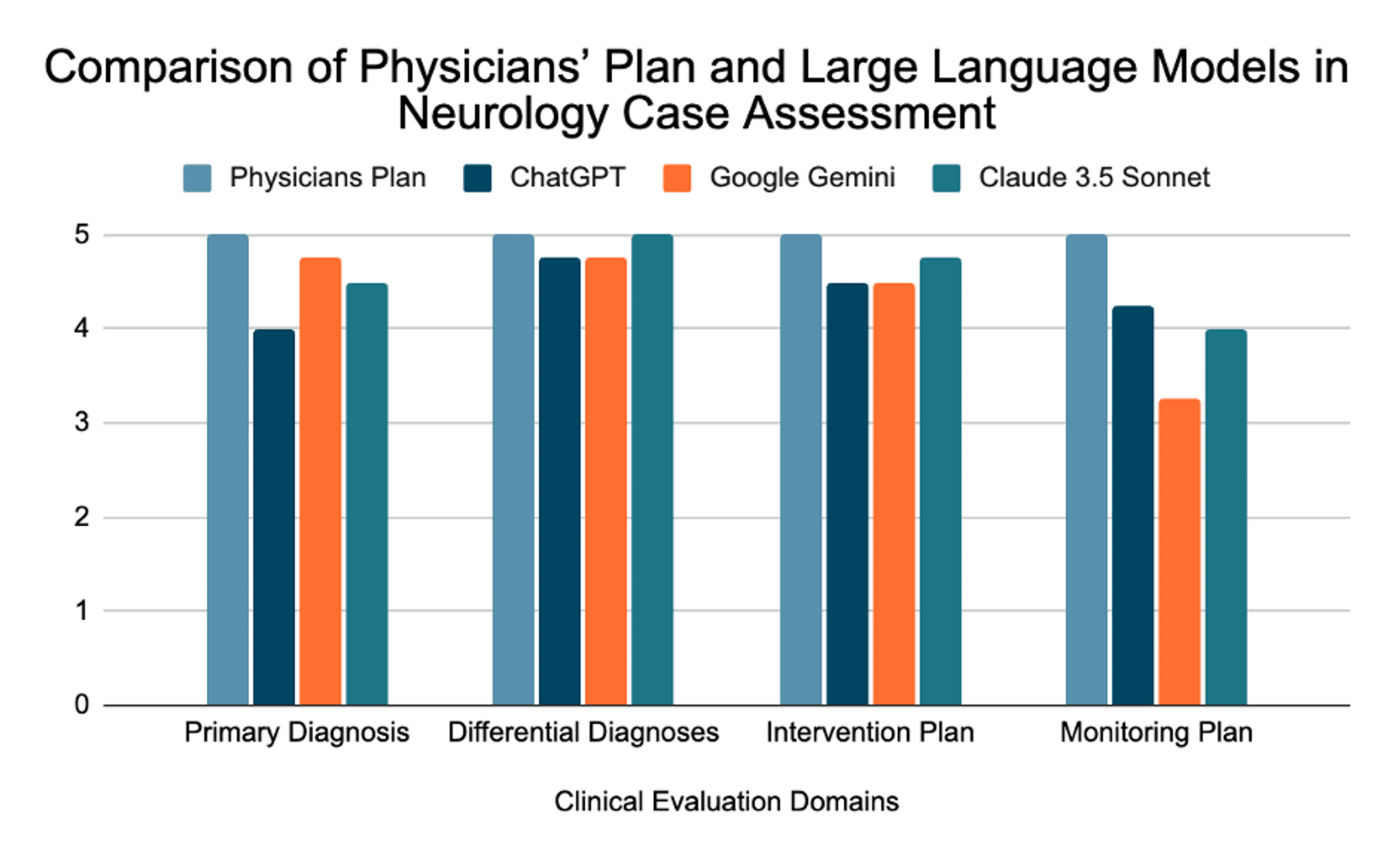
Comparison of Physician’s Plan and Large Language Models Across Clinical Evaluation Domains in a Neurology Case Grouped bar chart of mean domain scores (1–5; higher = better) assigned by four blinded, board-certified neurologists for a single complex case (acute GBS with chronic SEL). Models shown: Physicians Plan (reference), ChatGPT 4.0, Google Gemini, and Claude 3.5 Sonnet. Domains: primary diagnosis, differential diagnoses, intervention plan, monitoring plan. Bars represent means across raters; no error bars are plotted.

Four board-certified neurologists, blinded to the identity of each model, evaluated the assessment and management plans generated by ChatGPT 4.0, Google Gemini, and Claude 3.5 Sonnet. The physician’s plan, serving as the reference standard, scored 5.00 in all categories for a total score of 20. Scores for each LLM were assigned across four domains (primary diagnosis, differential diagnoses, intervention plan, and monitoring plan) and then summed to generate a total score for each evaluator-LLM pairing, as shown in Table 1. Mean scores for each model by category and total score are presented in Table 2 and illustrated in Figure 1.

**Table 1:** Individual Evaluator Scores for Large Language Models Across Diagnostic and Management Domains Blinded ratings by four board-certified neurologists of LLM outputs for a single complex case (acute GBS with chronic SEL). Four domains—primary diagnosis, differential diagnoses, intervention plan, and monitoring plan—were scored 1–5 (5 = best). Total Score sums domains (range 4–20; higher values indicate better overall performance). Analyses were descriptive only; computations were performed in R v4.4.3 (R Foundation, Vienna).

Evaluator	LLM	Primary Diagnosis	Differential Diagnoses	Intervention Plan	Monitoring Plan	Total Score
Evaluator 1	ChatGPT	3	4	3	2	12
Evaluator 1	Google Gemini	4	4	3	3	14
Evaluator 1	Claude 3.5 Sonnet	4	5	4	4	17
Evaluator 2	ChatGPT	3	5	5	5	18
Evaluator 2	Google Gemini	5	5	5	3	18
Evaluator 2	Claude 3.5 Sonnet	4	5	5	3	18
Evaluator 3	ChatGPT	5	5	5	5	20
Evaluator 3	Google Gemini	5	5	5	4	19
Evaluator 3	Claude 3.5 Sonnet	5	5	5	4	19
Evaluator 4	ChatGPT	5	5	5	5	20
Evaluator 4	Google Gemini	5	5	5	3	18
Evaluator 4	Claude 3.5 Sonnet	5	5	5	5	20

**Table 2:** Mean Scores of Large Language Models Compared to the Physician’s Plan Across Diagnostic and Management Domains This means four blinded neurologists using a 1–5 rubric for primary diagnosis, differential diagnoses, intervention plan, and monitoring plan. Total Score sums domains; Physician's Plan is the gold-standard reference. Descriptive analysis only (no hypothesis testing); computed in R v4.4.3

Large language model	Primary Diagnosis	Differential Diagnoses	Intervention Plan	Monitoring Plan	Total Score
Physician's Plan	5	5	5	5	20
ChatGPT	4.0	4.75	4.5	4.25	17.5
Google Gemini	4.75	4.75	4.5	3.25	17.25
Claude 3.5 Sonnet	4.5	5.0	4.75	4.0	18.5

Claude 3.5 Sonnet achieved the highest mean total score among the LLMs at 18.50, followed by ChatGPT at 17.50 and Google Gemini at 17.25. All three models accurately identified acute Guillain-Barré syndrome and recommended guideline-adherent therapies, including IVIG and plasmapheresis.

In primary diagnosis, Google Gemini had the highest mean score (4.75), followed by Claude 3.5 Sonnet (4.50) and ChatGPT (4.00). In differential diagnoses, Claude 3.5 Sonnet achieved a perfect mean of 5.00, while both ChatGPT and Google Gemini scored 4.75. For the intervention plan, Claude 3.5 Sonnet again led with 4.75, while ChatGPT and Google Gemini each scored 4.50. In monitoring and follow-up, ChatGPT had the highest mean (4.25), followed by Claude 3.5 Sonnet (4.00) and Google Gemini (3.25).

Reviewer observations were consistent with the quantitative results. Claude 3.5 Sonnet consistently produced the most complete and well-supported differential diagnoses and intervention plans. ChatGPT performed well in differential diagnoses and demonstrated its greatest strength in monitoring and follow-up recommendations, where it outscored both other models. Google Gemini showed the highest primary diagnostic accuracy but provided less detailed follow-up recommendations, resulting in its lowest score in the monitoring category. Despite these strengths, none of the LLMs matched the physician reference standard in addressing SEL-specific considerations or providing detailed, personalized longitudinal care.

Although no statistically significant differences were observed in total scores, the category-level analysis highlighted distinct performance patterns, with each model demonstrating unique strengths relative to the physician’s gold standard.

These descriptive findings illustrate that while all models demonstrated strong diagnostic accuracy and evidence-based management capability in this exploratory case-based study, domain-level variation and limited contextual reasoning highlight the continued need for physician oversight. 

## Discussion

Key findings

This study evaluated the ability of three LLMs (ChatGPT 4.0, Google Gemini, and Claude 3.5 Sonnet) to produce outputs with both diagnostic and therapeutic plans for a given patient case. Despite the neurological challenge provided, all three of the models correctly diagnosed acute GBS in the setting of chronic SEL. In addition, all of the LLMs provided treatment plans that were adherent to established treatment guidelines (IVIG, plasmapharesis). Claude 3.5 Sonnet performed at the highest level overall. However, there were no statistically significant differences amongst the performance of the LLMs (ANOVA p = 0.29). Consistent with prior literature, all models demonstrated strong performance on structured diagnostic tasks but variability in contextual reasoning. Monitoring/follow-up care was the weakest performance area for the LLMs overall when compared to the physician gold standard, suggesting better acute care recommendations but limited thoroughness in long-term management and rehabilitation plans.

Diagnostic performance and contextual understanding

In this patient case (Appendix), with considerable amounts of overlapping pathology, the LLMs managed to display contextual comprehension and identify the primary disease and cause of presenting symptoms. However, the plans included minimal incorporation of SEL-specific considerations (e.g., rehab modification) within the overall GBS management plan. This indicates a potential gap in patient-specific adaptation. Further, the physician provided a comprehensive, detailed follow-up plan, while the LLMs fell short with adequate but general recommendations that lacked specificity and contained omissions in timelines and personalization. Although this study did not compare LLM diagnostic performance to that of the physician (the physician’s diagnostic plan was used as the gold standard), other studies have suggested LLMs have statistically superior diagnostic accuracy when compared to human physicians in several contexts [8]. This is believed to be due to improved interpretation of information and pattern recognition in LLMs, rather than any difference in history taking or information acquisition [8]. Further, humans consistently display diagnostic bias. However, this study’s prompt did not include additional comorbidities (e.g., diabetes, hypertension) or gradations of disease severity, which are known to influence diagnostic reasoning. The absence of these confounders may have simplified the clinical scenario for the models and limited their applicability to more complex real-world presentations.

Physician burnout and physician shortage

One of the strongest arguments in support of increasing AI systems in healthcare is to combat the growing national physician shortage, as well as increasing rates of physician burnout. Particularly in neurology, an aging population presents an increasing demand for more neurologists. The shortage of neurologists in the U.S. has increased over the past decade and is expected to worsen, particularly in rural areas [9]. Similarly, another study showed that up to 60% of neurologists in the U.S. report at least one symptom of burnout [10]. Emerging studies suggest that LLM-based documentation and decision-support tools can reduce clinician workload by decreasing time spent on administrative and EHR-related tasks, thereby easing one contributor to physician burnout [11,12]. Although these systems have shown promise by documenting critical information discussed in patient encounters and expediting note writing, this study aimed to explore the ability of AI systems to assist with clinical decision-making, including diagnostic and treatment plans. More studies should be performed to explore the performance of AI in this specific context and whether it can be employed as a physician substitute, co-pilot, or helpful aid.

Health equity 

Although AI is easier to implement in a high-resource medical setting, it holds the most potential for improving care in low-resource settings. In small rural hospitals or community health clinics, specialists are often unavailable. In these settings, AI could transform the types of care that could be offered to underserved populations. An example of this already being used is the IDx-DR machine learning software program that utilizes AI to analyze retinal images and diagnose diabetic retinopathy [13]. It is being utilized in areas without available ophthalmologists and providing expertise to primary care physicians that can aid with diagnoses that may not otherwise be found [13]. Further, these low-resource areas often show extensive waiting periods to be seen by specialists, and AI can increase earlier detection for increased clinical outcomes. However, these scenarios represent situations in which AI and LLMs are aiding human physicians in diagnosis, rather than being the sole source of diagnosis. Our study displays limitations that still exist, and AI recommendations should be used with caution and at the discretion of a physician.

Strengths of this study

There are a few aspects that increase the validity of this study’s findings. First, there was significant ambiguity in the patient case. By incorporating two conditions with significant overlap, a complex, contextual challenge was created for the LLM. One in which its ability, or lack thereof, to personalize diagnostic and treatment plans would be displayed. Second, the blinding of the expert reviewers helped minimize evaluator bias so that their scoring was objective. Third, utilization of a rubric with predefined scoring criteria and definitions for each point assignment helped make scoring reproducible across reviewers. 

Limitations of this study 

The generalizability of this data is limited by using only a single case prompt. The LLMs may vary in performance depending on the type or complexity of presenting neurological conditions. While Claude 3.5 Sonnet consistently performed the highest in this case, the other LLMs could potentially show superiority in simpler cases with less multisystem involvement or fewer overlapping conditions. Another limitation is the use of only three LLM models. These results do not apply to all LLMs, and other AI systems may drastically differ in performance when attempting complex neurological care. Although the phrasing and length of the prompt for each LLM were identical in this study, different prompts and provided information would almost certainly have resulted in different outputs from each LLM. In the real world, there would be no standardized input for a given case. Rather, it would depend on what the LLM user decided to input. In addition, the plan created by the physician in the case report was utilized as the gold standard, but this plan only reflects that of a single expert’s approach. This approach may not align with other experts and clinical practices. Lastly, LLMs constantly evolve, so their performance in this study only represents their abilities at the time data was collected. 

The study also has several methodological limitations. Although the case prompt was written to be clear and neutral to minimize bias, it is not possible to prevent LLMs from having been previously exposed to similar cases during pretraining, and their internal training data cannot be controlled. The scoring rubric was developed specifically for this exploratory pilot study and was not formally validated. Inter-rater reliability was not calculated due to the small number of reviewers (N = 4), limiting the feasibility of meaningful κ or ICC analysis. Future studies should include rubric validation, reliability testing, and expanded reviewer cohorts to strengthen methodological rigor.

Future directions

Future research should investigate LLM performance when delivering a diverse array of cases with varying neurological complexity and multisystem involvement. This study included an acute presentation, but future studies could include both acute and chronic presentations, with an emphasis on the use of LLMs for guiding longitudinal care. Studies should also be performed to explore LLM performance in other specialties, rather than neurological cases, to help highlight the specific strengths and weaknesses of each LLM and increase the generalizability of the data. This information could help guide policy regarding AI use in clinical settings and its appropriate role in relation to physician oversight. Effort should be made to improve the ability of LLMs to create detailed, personalized treatment plans that do not simply follow standard guidelines, especially in patients with several comorbid conditions. Studies should also explore the variation in LLM performance based on different iterations of prompt inputs from the same patient case. There should be real-world implementation studies that include LLMs in clinical workflows and analyze patient outcomes to measure their practical value. Finally, these models should be provided with large, diverse datasets and trained to minimize bias to maximize patient safety and promote health equity in a variety of healthcare settings.

Future studies should also evaluate LLM performance in cases that include multiple comorbidities, atypical disease presentations, or severity gradients, as these factors more accurately reflect real-world diagnostic challenges and may reveal additional model limitations.

Because LLMs evolve rapidly, these findings represent a time-bound snapshot of performance; future work should include version-specific reporting and periodic re-evaluation using standardized benchmarks to ensure ongoing validity as models are updated.

## Conclusions

LLMs displayed diagnostic accuracy and the ability to generate guideline-adherent treatment recommendations for a complex neurological case involving GBS in the setting of SEL. Although model performance varied, all LLMs demonstrated important limitations in contextual reasoning and personalized, longitudinal care planning, reinforcing the need for physician oversight. Given the single-case design and limited statistical power, these findings should be interpreted as exploratory and hypothesis-generating rather than definitive, and broader conclusions about LLM performance in neurology will require larger, more diverse clinical evaluations. Going forward, improving LLM capacity to incorporate comorbidities, disease severity, and patient-specific factors will be essential for safe clinical use. Developing standardized evaluation frameworks, incorporating version-specific benchmarking, and integrating LLMs into supervised clinical workflows may support their future role as reliable decision-support tools. While LLMs show promise, they are not substitutes for clinical expertise; continued refinement, validation, and real-world testing are necessary before broader implementation in neurological care.

## Appendices

Categories and point allocation

Primary Diagnosis (1-5 Points)

5 points: Accurately identifies Guillain-Barré Syndrome (GBS) with detailed, clinician-level reasoning tied directly to case findings.

4 points: Correctly identifies GBS with strong reasoning but omits minor supporting details.

3 points: Identifies GBS but provides incomplete or partially inaccurate reasoning.

2 points: Suggests GBS but with weak or unclear reasoning and minimal evidence.

1 point: Misidentifies or completely omits the primary diagnosis.

Differential Diagnoses (1-5 Points)

5 points: Includes at least four well-supported differential diagnoses with clear, logical rationale and evidence for each.

4 points: Provides four differentials but with minor gaps in reasoning or slightly weaker support for one.

3 points: Includes three differentials with adequate rationale but some noticeable gaps or omissions.

2 points: Provides only one or two plausible differentials with limited reasoning or evidence.

1 point: Fails to provide adequate or accurate differential diagnoses.

Intervention and Treatment Plan (1-5 points)

5 points: Cmprehensive plan covering immediate interventions (e.g., ICU admission, respiratory support), appropriate treatments (e.g., IVIG dosing), and rationale closely aligned with the clinician’s recommendations.

4 points: Provides a reasonable and mostly accurate plan with minor omissions or a small error.

3 points: Covers basic interventions and treatment but lacks depth, includes notable omissions, or contains multiple inaccuracies.

2 points: Presents a vague or incomplete plan with significant gaps or inconsistencies.

1 point: Fails to provide an appropriate or safe intervention and treatment plan.

Monitoring and Follow-Up (1-5 points)

5 points: Detailed, specific plan addressing key areas such as respiratory monitoring, neurological assessment, and long-term follow-up care, aligned with clinical standards.

4 points: Provides an adequate plan with minor omissions or less detailed recommendations.

3 points: Minimal plan with noticeable gaps or insufficient specificity.

2 points: Presents vague or unclear monitoring strategies, omitting critical elements.

1 point: Fails to address monitoring and follow-up effectively or at all.

Total Score: 20 Points

18-20: Excellent - Matches or exceeds clinician-level detail, reasoning, and comprehensiveness.

14-17: Good - Aligns well with the clinician’s plan but has some omissions or lacks depth in one or two areas.

10-13: Fair - Covers basic elements but lacks detail, contains gaps, or includes inaccuracies in multiple areas.

4-9: Poor - Fails to meet clinical standards, with significant omissions or unsafe recommendations.

LLM Output Evaluation Score Sheet

Scoring Criteria: Instructions: For each category, assign a score from 1 to 5 for each LLM. Use the comments section to justify scores and provide detailed observations.

Primary Diagnosis (1-5 Points)

5: Accurately identifies GBS with detailed reasoning tied directly to case findings.

4: Correctly identifies GBS but omits minor supporting details.

3: Identifies GBS but provides incomplete or partially inaccurate reasoning.

2: Suggests GBS with weak or unclear reasoning and minimal evidence.

1: Misidentifies or omits the primary diagnosis.

Differential Diagnoses (1-5 Points)

5: Includes at least four well-supported differentials with clear, logical rationale.

4: Provides four differentials with minor gaps in reasoning or slightly weaker support.

3: Includes three differentials with adequate rationale but noticeable gaps.

2: Provides one or two plausible differentials with limited reasoning or evidence.

1: Fails to provide adequate or accurate differential diagnoses.

Intervention and Treatment Plan (1-5 Points)

5: Comprehensive plan covering immediate interventions, appropriate treatments, and rationale.

4: Reasonable and mostly accurate plan with minor omissions or small errors.

3: Covers basic interventions but lacks depth, includes omissions or inaccuracies.

2: Vague or incomplete plan with significant gaps or inconsistencies.

1: Fails to provide an appropriate or safe intervention plan.

Monitoring and Follow-Up (1-5 Points)

5: Detailed, specific plan addressing respiratory, neurological, and long-term follow-up.

4: Adequate plan with minor omissions or less detailed recommendations.

3: Minimal plan with noticeable gaps or insufficient specificity.

2: Vague or unclear strategies, omitting critical elements.

1: Fails to address monitoring and follow-up effectively.

Case Presentation

A morbidly obese 43-year-old male was initially seen for bilateral hand and leg numbness and very mild weakness. At the time of examination, he only demonstrated mildly decreased sensation in his hands and feet and subjective extremity weakness. He complained of not being able to walk, but he had walked in unassisted during that first visit. He had no known medical problems except possible “pre-diabetes,” hypertension, and anxiety. He was not on any medications. He was a non-smoker and had never used any intravenous drugs. He had not had any instrumentation to his back, and he had not fallen or experienced any back trauma. A broad workup was done, including general lab testing, all of which was normal. He was discharged home with specific instructions to follow up with his primary care doctor to get a referral to neurology, as his differential diagnosis included diabetic neuropathy or an underlying chronic neurologic condition such as multiple sclerosis. The patient did see his internist, but unfortunately, was simply referred back to the emergency room for a neurology evaluation. On this second visit, he explained that he was progressively losing the ability to use his arms and legs and that he was unable to walk. He was unable to get himself to sit on the toilet, although he reported normal bowel and bladder function. He did have to come in via wheelchair. During this second visit, the further history of the present illness was obtained. A few weeks before the presentation, the patient had visited the Dominican Republic, where he caught a viral illness and developed multiple cold sores in his mouth. When he returned from this trip, he experienced 1 week of diarrhea, which was then followed by weakness in his arms and legs. On review of systems, he denied fevers, chills, chest pain, shortness of breath, nausea, vomiting, diarrhea, abdominal pain, back pain, headache, bowel or bladder incontinence, or dysuria. His vital signs were a temperature of 98°F, a pulse of 84 beats per minute, a respiration rate of 16 breaths per minute, a blood pressure of 122/91 mmHg, and 100% for his oxygen saturation on room air with a BMI of 46.3. On his physical exam, the patient had significant bilateral lower extremity weakness, which was new compared to the prior visit. It was slightly worse on the right than the left. He was completely unable to lift the leg on the right, and even when it was lifted up for him, it dropped to the bed. On the left, he could not sustain lifting his leg, but he was able to have it gently come down to the bed. His National Institutes of Health Stroke Scale (NIHSS) was 5 due to these motor deficits. He had a weak handgrip with a score of 3/5 in both hands. He had a normal finger-to-nose bilaterally. He had questionably diminished reflexes in the lower extremities and intact reflexes in the upper extremities. His sensation was intact in both his lower extremities. He had fine motor movement difficulty in both his hands. Cranial nerve examination was normal. There was no dystonia, no fasciculations, no myoclonus or tremor, as well as no open wounds in the back and no step-offs or deformities on the exam. Rectal examination revealed normal tone and normal sensation of the anal area. The patient’s laboratory analysis was unremarkable, including a negative COVID-19 test. Computed tomography (CT) imaging of the cervical and thoracic spine revealed no osseous injury or significant spondylosis. However, a CT of the lumbar spine revealed an increased volume of epidural fat visualized from L3 through S1, consistent with spinal epidural lipomatosis. Mild-to-moderate left foraminal stenosis at L5-S1 was also noted (Figure 1). Eventually, he developed absent patellar, Achilles, and plantar reflexes bilaterally, but with 2+ reflexes in the bilateral biceps, triceps, and brachioradialis. MRI was unable to be performed due to the patient’s body habitus. The patient refused a lumbar puncture.
